# Fastidious Operating

**Published:** 1888-01

**Authors:** 


### FASTIDIOUS OPERATING.

If there be any one thing which a dentist should cultivate, it is delicacy and lightness of touch. Some whom we have known go at their work like a miner with his pickaxe. They are rough, harsh, and their hand, whether with the excavator, the plugger, or engaged in adjusting the various appliances of our art, is ever heavy. Their arm always rests burthensomely upon the patient's head, their fingernails are continuously digging into tender tissues, and there is a coarseness and a clumsiness about all their operations that marks an unpardonable heedlessness of the comfort of the patient. There are few things which so forcibly commend an operator to those under his care as tenderness, and even daintiness, in regarding their sensibilities. The engine bur should be directed as if it were a sentient thing, and napkins should be used as if they were a spontaneous production.

